# Supplementary material for: The Occurrence and Dietary Exposure Assessment of Mycotoxins, Biogenic Amines, and Heavy Metals in Mould-Ripened Blue Cheeses
Source: Foods. 2020 Jan 16;9(1):93. doi: 10.3390/foods9010093 (PMC7023506; doi:10.3390/foods9010093)
Supplement: Supplementary file 1 [file foods-09-00093-s001.zip › Supplementary material 1.docx]

**The occurrence and dietary exposure assessment of mycotoxins, biogenic amines, and heavy metals in mould-ripened blue cheeses**

Reinholds I. et al.

**Supplementary data**

**Figure S1**. The stability of biogenic amine derivates during the sequence of 20 injections (test conditions: blank sample spiked with a standard mixture at 50 mg kg^-1^ concentration).


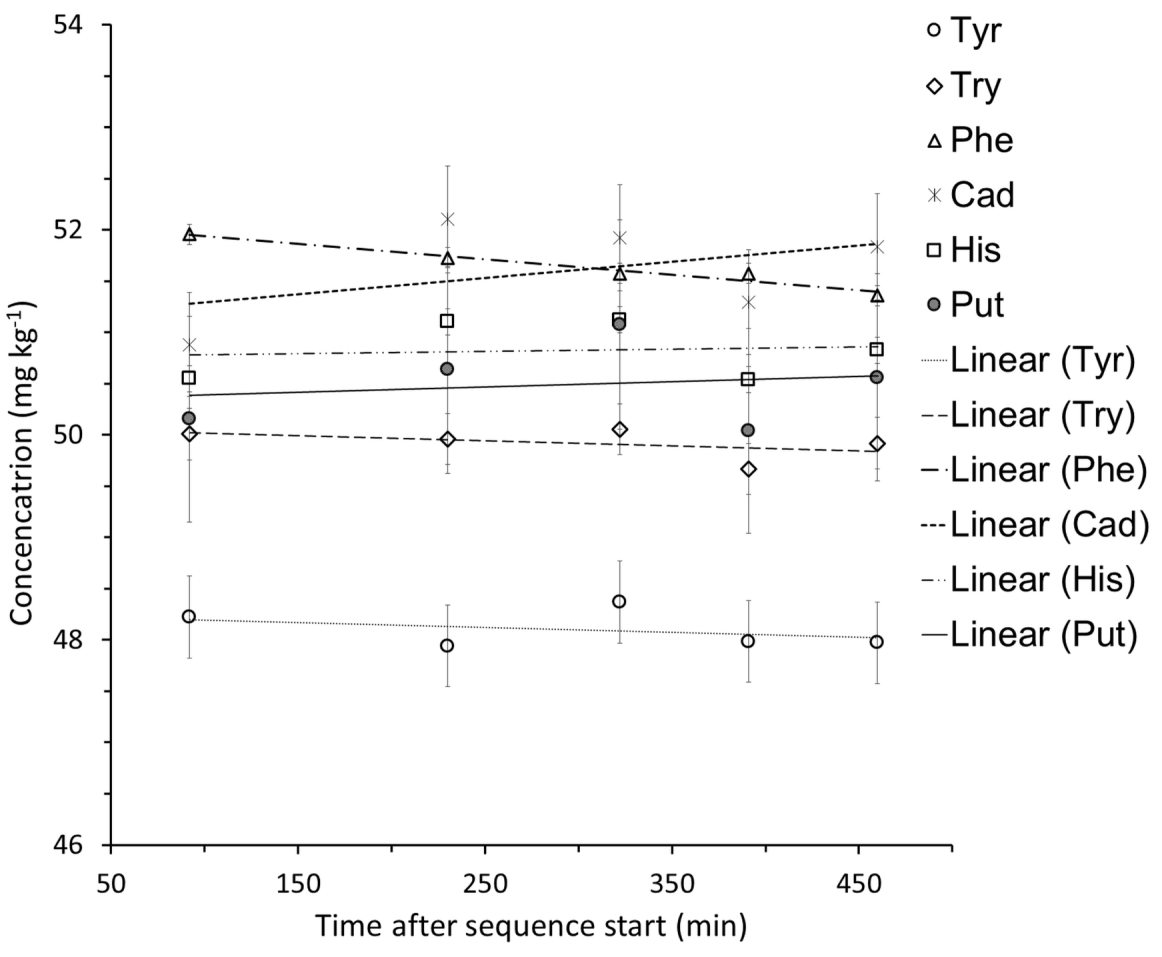


**Table S1.** Validation data of LC-MS/MS method.

| **Mycotoxin** | **Linear range**  **(µg kg^-1^)** | **Linearity (R^2^)** | **LOD**  **(µg kg^-1^)** | **LOQ**  **(µg kg^-1^)** | **Recovery (%)**  **(mean, n=36)** | **Intra-day RSD (%) (mean, n=36)** | **U (%)** |
| --- | --- | --- | --- | --- | --- | --- | --- |
| PA | 10–130 | 0.993 | 0.09 | 0.28 | 109 | 10 | 20 |
| AFM_1_ | 0.02–0.12 | 0.996 | 0.004 | 0.012 | 100 | 9 | 18 |
| ROQ C | 5.0–500 | 0.999 | 0.12 | 0.37 | 94 | 5 | 9.6 |
| AFB_1_ | 1.0–25 | 0.993 | 0.05 | 0.15 | 106 | 5 | 10 |
| MPA | 5.0–500 | 0.999 | 0.47 | 1.41 | 117 | 4 | 8 |
| OTB | 1.0–25 | 0.997 | 0.06 | 0.18 | 108 | 5 | 9 |
| CVD | 5.0–500 | 0.995 | 0.17 | 0.52 | 96 | 16 | 32 |
| SBL | 1.0–20 | 0.991 | 0.04 | 0.13 | 101 | 3 | 6 |
| OTA | 1.0-25 | 0.995 | 0.30 | 1.00 | 93 | 13 | 26 |
| CIT | 5.0-100 | 0.998 | 0.02 | 0.07 | 99 | 8 | 17 |
| PNA | 1.0-25 | 0.993 | 0.86 | 2.58 | 100 | 18 | 36 |
| ENN B | 1.0-20 | 0.995 | 0.005 | 0.014 | 103 | 12 | 24 |
| ENN B_1_ | 1.0–20 | 0.995 | 0.05 | 0.16 | 95 | 10 | 20 |
| BEA | 1.0–25 | 0.997 | 0.08 | 0.25 | 96 | 29 | 58 |
| ENN A | 1.0–20 | 0.991 | 0.07 | 0.22 | 91 | 12 | 24 |
| ENN A_1_ | 1.0–20 | 0.996 | 0.04 | 0.13 | 107 | 13 | 27 |

LOD: level of detection, LOQ: level of quantification, RSD: relative standard deviation, U: measurement uncertainty

**Table S2.** Validation data of LC-PAD method.

| **Biogenic amine** | **Added concentration**  **(mg kg^-1^)** | **Linearity (R^2^)** | **LOD**  **(mg kg^-1^)** | **LOQ**  **(mg kg^-1^)** | **Recovery (%)**  **(mean, n=15)** | **RSD (%) (mean, n=15)** | **U (%)** |
| --- | --- | --- | --- | --- | --- | --- | --- |
| Try | 1 | 0.999 | 0.19 | 0.38 | 99.2 | 9.3 | 26.7 |
|  | 20 |  |  |  | 106.4 | 8.0 |  |
|  | 58 |  |  |  | 88.3 | 9.3 |  |
| Put | 1 | 0.996 | 0.12 | 0.25 | 95.0 | 3.9 | 26.8 |
|  | 20 |  |  |  | 98.0 | 5.1 |  |
|  | 58 |  |  |  | 87.7 | 10.4 |  |
| Phe | 1 | 0.996 | 0.26 | 0.52 | 101.4 | 4.9 | 27.4 |
|  | 20 |  |  |  | 91.1 | 6.8 |  |
|  | 58 |  |  |  | 80.7 | 7.8 |  |
| Cad | 1 | 0.996 | 0.09 | 0.18 | 95.6 | 7.8 | 27.4 |
|  | 20 |  |  |  | 96.6 | 8.7 |  |
|  | 58 |  |  |  | 83.3 | 10.4 |  |
| His | 1 | 0.997 | 0.19 | 0.37 | 101.9 | 9.6 | 26.8 |
|  | 20 |  |  |  | 96.4 | 9.2 |  |
|  | 58 |  |  |  | 84.0 | 11.2 |  |
| Tyr | 1 | 0.996 | 0.48 | 0.96 | 94.8 | 14.3 | 32.1 |
|  | 20 |  |  |  | 98.7 | 15.7 |  |
|  | 58 |  |  |  | 88.4 | 8.4 |  |

LOD: level of detection, LOQ: level of quantification, RSD: relative standard deviation, U: measurement uncertainty

**Table S3.** Validation data of ICP-MS method.

| **Element** | **Linearity (R^2^)** | **LOD**  **(mg kg^-1^)** | **LOQ**  **(mg kg^-1^)** | **Recovery (%)** | **RSD (%)** | **U (%)** |
| --- | --- | --- | --- | --- | --- | --- |
| Al | 0.9998 | 0.320 | 1.07 | 93 | 8.47 | 21.5 |
| Mn | 0.9999 | 0.083 | 0.276 | 110 | 6.36 | 23.7 |
| Fe | 0.9998 | 1.57 | 5.24 | 86 | 3.12 | 28.2 |
| Co | 0.9998 | 0.037 | 0.123 | 102 | 8.05 | 16.4 |
| Ni | 0.9999 | 0.112 | 0.373 | 109 | 9.88 | 35.7 |
| Cu | 0.9997 | 1.97 | 6.55 | 107 | 3.94 | 15.3 |
| Zn | 0.9999 | 5.30 | 17.7 | 110 | 4.25 | 21.4 |
| As | 1.0000 | 0.013 | 0.042 | 110 | 4.17 | 21.5 |
| Se | 0.9999 | 0.042 | 0.139 | 107 | 13.01 | 29.6 |
| Mo | 0.9997 | 0.046 | 0.154 | 115 | 7.81 | 34.3 |
| Cd | 0.9998 | 0.002 | 0.008 | 108 | 9.42 | 24.8 |
| Sn | 1.0000 | 0.023 | 0.077 | 115 | 10.77 | 22.0 |
| Pb | 0.9998 | 0.007 | 0.023 | 107 | 0.78 | 14.0 |

LOD – level of detection, LOQ – level of quantification, RSD – relative standard deviation, U – measurement uncertainty
